# Organisation of health services for the delivery of primary health care in the WHO African region: a future perspective

**DOI:** 10.1016/j.lanprc.2026.100122

**Published:** 2026-04

**Authors:** Humphrey Karamagi, Anabay Mamo, Benson Droti, Benjamin Tsofa, Sokona Sy, Solyana Kidane, Nelida Cabral, Juliet Nabyonga, Prosper Tumusiime

**Affiliations:** a WHO Country Office, Juba, South Sudan; b WHO Regional Office for Africa, Brazzaville, Republic of the Congo; c Health Systems and Research Ethics Department, KEMRI-Wellcome Trust Research Programme, Kilifi, Kenya; d WHO Country Office, Maputo, Mozambique; e WHO Country Office, Windhoek, Namibia; f Kampala, Uganda

## Abstract

Despite decades of investment in health systems across the WHO African region, population health outcomes remain suboptimal. The region faces evolving challenges, including demographic shifts, emerging health threats, and persistent inequalities. Current health-service delivery models are misaligned with anticipated future health demands, necessitating a reimagined operational framework grounded in a revitalised primary health-care approach. In this Viewpoint, we draw on expert consensus from professionals across 19 countries using the nominal group technique and Delphi-style rounds. Experts were organised into thematic “policy laboratories” focusing on primary care, hospitals, and oversight. Expert insights were collected through structured questionnaires, thematic analysis, and iterative validation, culminating in a 5-day consensus workshop. Three key constructs emerged for future health-service organisation: (1) primary care units as integrated networks delivering first point-of-care interventions; (2) hospitals redefined to include training, research, and clinical governance roles; and (3) oversight structures with decentralised, participatory, and evidence-informed decision-making capacities. The proposed model emphasises person centredness, functional integration, and digital innovation to enhance system responsiveness and resilience. The future of health-service delivery in Africa lies not in replacing existing structures, but in repurposing and realigning them to meet population health needs. Incremental reforms, supported by digital tools, essential health packages, and rationalised service-provision modalities, can enable countries to build resilient, people-centred health-care systems. National and subnational leadership, supported by regional and global partners, is essential for driving this transformation.

## Introduction

African health systems have evolved through a layered history shaped by global and local forces. Following colonisation, most countries inherited centralised, top-down structures designed for administrative control rather than community engagement.^1^ The 1978 Alma-Ata Declaration and the Health for All agenda marked a turning point, introducing primary health care (PHC) as a community-oriented model emphasising prevention, equity, and accessibility.^2^ However, structural adjustment programmes of the 1980s and 1990s shifted priorities towards cost recovery and reduced public-sector spending, weakening PHC investments.^3^, ^4^, ^5^, ^6^ Nowadays, African health systems reflect this complex legacy, with external priorities and fragmented approaches influencing governance and service delivery.

**Search strategy and selection criteria**

This Viewpoint is informed by structured policy laboratories that explored the implementation of the revitalised primary health-care approach in the WHO African region through expert consensus from professionals across 19 countries, involving policy makers, implementers, and technical experts from the region, using the nominal group technique and Delphi-style rounds. In addition, we performed a literature review to support the conceptual framing and interpretation of findings. Relevant evidence, including peer reviewed articles, policy documents, and normative guidance, were identified by searching PubMed, Cochrane Library, Google Scholar, WHO institutional repositories, and reference lists of key publications from Sept 12, 1978, to June 25, 2025. Search terms included combinations of primary health care, revitalised primary health care, health systems, health-system functionality, services integration, people-centred care, hospitals, health facilities, district, Africa, and WHO African region: “primary health care”, “primary healthcare”, “revitalised primary health care”, “revitalized primary health care”, “health system”, “health systems”, “health system strengthening”, “health system functionality”, “integrated health services”, “service integration”, “health services integration”, “people-centred care”, “people-centered care”, “district health system”, “district health systems”, “hospitals”, “health facilities”, “service delivery”, “WHO African Region”, and “Africa”. Publications in English, French, and Portuguese—WHO working languages—were included to capture the evolution of the primary health-care approach. Evidence identified via the search guided the framing of the Viewpoint focus areas.

Population health outcomes in the countries of the WHO African region continue to lag behind those of other regions worldwide, despite a sustained focus on the region by global actors.^7^ Since the formal adoption of the PHC approach in 1978, local and global actors have made substantial investments to close this persistent gap in health outcomes.^8^ African health systems remain fragile, and uptake of health services by potential beneficiaries is suboptimal. As a result, there is a perceived gap between health gains and investments made.^8^

The future in which health systems and services need to function is becoming more challenging. The region is grappling with changing sociodemographics, emerging and re-emerging health needs and threats, persisting inequalities, and environmental challenges.^9^^,^^10^ However, there have been achievements in tackling specific diseases such as malaria, HIV/AIDS, diarrhoeal diseases, and lower respiratory infections, better investments in specific health-system areas such as health workforce, medical products and supply systems—health infrastructure, and health-information systems, and effective engagements with communities that have driven successes in several countries such as Guinea, Liberia, and Sierra Leone.^11^ Substantial opportunities exist to improve health in the region, such as expanding economic opportunities, the digital revolution, and private sector engagements.^12^

**Figure 1:**
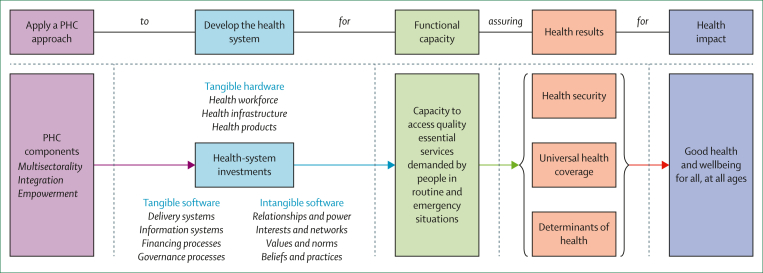
Interlinkages among global health directions The figure illustrates the authors’ interpretation of the PHC approach, and its linkage to universal health coverage and the Sustainable Development Goals. The figure is adapted from WHO and UNICEF’s PHC measurement framework and indicators.^14^ PHC=primary health care.

The current approach to developing health systems in the WHO African region is not fit-for-purpose for the future of health services, as needs are changing but the strategic design and focus are not evolving. The WHO African region has adopted a framework for health-system development to guide what investment, and the revitalised PHC approach directs how these investments should be implemented.^13^ Six interlinked concepts currently guide health priorities across countries in the region (figure 1).^14^

Operationalising health systems and services is essential to ensure adequate health outcomes in the region.^15^^,^^16^ Planning, organisation, management, and monitoring of service provision are necessary to define at the operational level, in line with the renewed paradigms and evolving health context.^15^^,^^17^^,^^18^

In this Viewpoint, we explore the future of health-service delivery in Africa, proposing strategies to structure health services at the operational level. We aim to provide operational guidance for national decision makers on designing subnational services that enable planning, organisation, delivery, management, and monitoring of service delivery in line with future needs. We focus on subnational management and oversight, hospital services, and primary care services as constructs of the operational level of health-service delivery.

## Approach to exploring the future of service delivery in the WHO African region

Based on the literature search, we classified health-service delivery functions into three key institutional structures: primary care institutions, which provide targeted services; hospitals, which provide comprehensive services; and oversight institutions, which coordinate service delivery. We explored the future of health-service delivery in three policy laboratories, each focusing on one of these institutional structures.

We sought structured guidance from experts in the region to understand their views on the future roles of each of the three institutional structures. Such insights from the experts aimed to enrich the quality of views and inputs. We applied qualitative methods, namely, the nominal group technique, to generate ideas from the experts and Delphi-style consensus rounds to build agreement within each of the three institutional structures.^19^^,^^20^ The process was carried out in five steps.

In step one, we identified experts representing the three policy laboratories from all the countries in the WHO African region, covering primary care, hospitals, and oversight functions within the subnational or district health systems. The experts were selected using purposive sampling.^21^ Experts were identified by health planning directors in both Ministries of Health and WHO country offices. Four categories of experts were included in each laboratory: (1) experienced professionals with over 10 years of relevant expertise, (2) practitioners currently working in the specific policy laboratory focus structure, (3) academics undertaking analytical work related to the specific policy laboratory focus structure, and (4) advocates who are promoters of the specific policy laboratory focus structure. 58 experts from 19 countries were enrolled: 16 in the primary care policy laboratory, 19 in the hospital policy laboratory, and 23 in the oversight policy laboratory (appendix pp 2–3).

In step two, we distributed a semistructured questionnaire to all identified experts (appendix p 3) to collate their views on the future of health-service delivery in Africa. The experts were provided with appropriate contextual information and guided through the tool during a virtual meeting (appendix pp 4–9). We solicited each expert’s views on four attributes of their policy laboratory: (1) practices, (2) bottlenecks, (3) future options, and (4) aspirations.^22^ The responses were collated and consolidated into a single summary report for each policy laboratory and shared with the experts for additional views or inputs.

In step three, we collated and summarised the inputs from each expert within a policy laboratory.^22^ These inputs were thematically analysed, and a menu of options was extracted under four thematic areas: (1) a definitional summary of the policy laboratory, (2) services the policy laboratory should provide, (3) core inputs needed, and (4) prerequisites for preparing the policy laboratory area for transformation. This summary was guided by both existing literature and expert knowledge of the authors. Consistent with the nominal group technique principles, step three prioritised inclusivity over consensus, incorporating all options identified by the experts.

In step four, we shared the compiled summary options generated by the authors with all 58 experts involved in each specific policy laboratory for review and validation. The experts were asked to share their views on any aspects of the summary with which they disagreed.

Step five comprised a 5-day in-person meeting of the experts to finalise consensus on the options for the future of health-service delivery. 31 of the 58 experts participated in this meeting, including 8 of 16 experts from the primary care policy laboratory, 8 of 19 experts from the hospital policy laboratory, and 15 of 23 experts from the oversight policy laboratory (appendix pp 2–3). The nominal group technique was used to identify areas of divergence, followed by the Delphi technique to build consensus on these points. Each laboratory reviewed the summary document, with every expert indicating their level of agreement with its content. Then, the experts from the three policy laboratories convened in one plenary session to deliberate on areas where there was no consensus. The first question relating to definition of the policy laboratory area was most contentious, requiring three Delphi rounds to achieve consensus, whereas the other three questions achieved consensus within one round. For subsequent rounds, consensus on question 1 was facilitated by the responses already agreed upon for questions 2–4. To ensure sufficient representation and incorporate broader views, consolidated inputs and strategic recommendations from each group were jointly reviewed in plenary sessions.

Therefore, the emerging issues presented represent the consolidated and agreed views of the diverse group of experts. These issues, together with our implicit knowledge of the region, form the basis for the future perspectives outlined in this Viewpoint.

## Emerging issues from the policy laboratories

### Contextual issues affecting health-service delivery

Health-system performance in the WHO African region varies across countries^7^ (appendix p 10), with strengths, weaknesses, opportunities, and threats observed in most countries. Among the strengths, there is consensus on the need for universal health coverage as a common result. Service coverage, a measure of use of essential services, is steadily improving in most countries, with the regional average score (out of 100) increasing from 23 in 2000 to 44 in 2021, and approaching 50 in 2024.^23^ New essential interventions are continuously being introduced in health systems in the region, including second dose of measles-containing vaccine in 41 countries, rotavirus vaccine in 38 countries, pneumococcal vaccine in 40 countries, and malaria vaccine in 17 countries.^24^, ^25^, ^26^ Investments in health systems have improved, with the health workforce growing at an annual rate of 13% since 2018.^27^^,^^28^ Increased communication and interconnectedness in the region have accelerated knowledge exchange and created opportunities for the use of emerging health technologies.

However, several weaknesses persist across health systems in the region. Health sectors grapple with emerging and re-emerging shock events. For example, zoonotic diseases increased by 63% during 2012–22 compared with 2001–12, along with persistent outbreaks of meningitis, cholera, Ebola disease, measles, poliomyelitis, yellow fever, and COVID-19.^9^ Health systems in countries in the region face shortages of staff, infrastructure, and medical products relative to the services they are expected to provide, a situation compounded by governance, financing, and information challenges.^7^ Health inequalities remain intractable both within and between countries and across interventions in the region.^29^^,^^30^ Regarding opportunities, the technological revolution offers new possibilities for innovative approaches to deliver and manage health services, if well channelled.^31^, ^32^, ^33^ Additionally, most countries can still benefit from the demographic dividend, as a large population of individuals aged 15–24 years provides opportunities for innovation and new ways of addressing health challenges.

Considerable threats impact the region, and many countries still face security, environmental, political, and social events that destabilise both the delivery and use of health care.^34^^,^^35^ Such instability directly impacts the ability of health systems to anticipate, adopt, adapt, and transform in response to ensure the sustainability of services.^36^, ^37^, ^38^

The present context provides a strong foundation for understanding how health-service delivery should change and be structured to meet future health needs. African countries also recognise the need for these changes, with 18 countries proposing six areas for improvement: (1) defining essential health services and increasing their availability, (2) increasing service coverage and targeting hard to reach populations, (3) providing financial risk protection, (4) improving user satisfaction with health services, (5) improving health security, and (6) expanding coverage of health-related sector services.^39^

**Figure 2:**
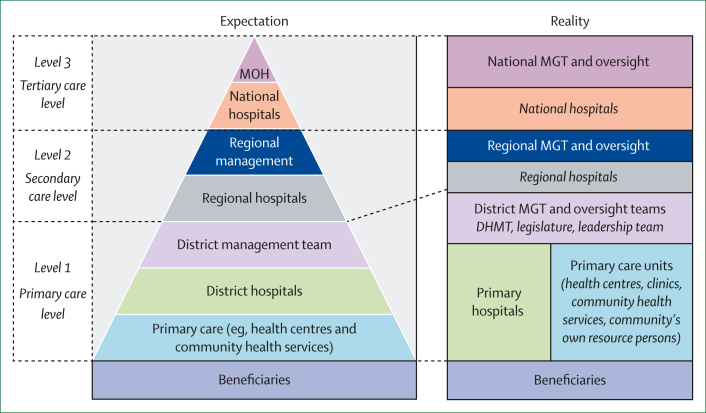
Expected and actual organisation of health services in the WHO African region The figure presents the authors’ consolidation of health-system concepts from the region. Resource persons are community members with specific skills that could be useful in supporting health-care activities. These persons could be formally trained (eg, community health workers) or untrained (eg, grandmothers or caregivers). MOH=Ministry of Health. MGT=management. DHMT=district health management team.

The situation is compounded by the scarcity of effective organisation of service delivery across the region. Although health systems are often described as operating in a pyramid structure, this model rarely reflects reality (figure 2). Figure 2 consolidates experiences from African countries drawn from the policy laboratories, insights generated through our literature search, and the practical experiences of public health professionals involved in developing this Viewpoint. The figure shows that service delivery is frequently top-heavy, with substantial development at higher levels and substandard functionality at the primary care level. Priority setting is often fragmented and programme driven rather than integrated. Although the PHC approach calls for beneficiaries to access services primarily at the primary care level, with investments and oversight focused there, actual service-use patterns are mixed. Beneficiaries access a range of services at both primary care facilities and hospitals, which are characterised by overlapping roles and weak referral linkages. Furthermore, health-system organisation varies considerably across countries and is influenced by governance arrangements, financing models, and the roles of non-state actors, including non-governmental organisations, private providers, and traditional health systems. These variations show the need for adaptable models that consolidate good practices, promote integration, and address fragmentation.

### Person centredness: a core focus for the future of health-service delivery

The effectiveness of future health-operational activities depends directly on how the operational level is organised and managed. Given the stated needs and context, health services should be refocused around the needs and expectations of beneficiaries, rather than primarily around diseases. This approach implies that all actions within the health system and health services should be designed based on how individuals would benefit. Services should be planned, organised, monitored, and reviewed based on their comprehensive impact on targeted individuals’ health and wellbeing. Health sectors should respond to the legitimate needs of individuals across all age groups, from newborns to older adults, and across the continuum of care, encompassing promotion, prevention, diagnosis, treatment, rehabilitation, and palliative interventions.

Health-service delivery institutions at both primary care and hospital levels, along with oversight capacities, need to be organised to address the legitimate needs of individuals and families. Organisationally, health-service delivery institutions should have six semi-autonomous units, irrespective of their level: (1) outpatient unit, (2) inpatient unit, (3) operative unit, (4) investigative unit, (5) specialised clinic unit, and (6) oversight unit. The scope and focus of each unit will differ substantially between primary care and hospital levels. In line with the revitalised PHC approach, primary care units (PCUs) serve as the first point-of-care, whereas hospitals provide comprehensive and specialised services. In practice, on-site provision within each domain varies by level and context. Primary care institutions should prioritise strengthening locally feasible functions and complement these through networked arrangements, shared resources, and robust referral systems for domains beyond their capacity. This approach emphasises continuity of care through incremental improvements rather than wholesale structural changes, building on existing good practices and adaptable models. The added value lies in consolidating effective practices from diverse systems into a coherent, future-oriented framework that promotes integration, person-centredness, and resilience, without imposing unrealistic infrastructure requirements.

### PCUs

PCUs serve as the initial point of contact with the health system; however, they are currently underperforming because of fragmentation and poor integration. Services are often delivered through vertical programmes, such as HIV or immunisation initiatives, which undermine continuity and person-centred care. Inadequate infrastructure and staff shortages further restrict the scope and quality of services, and weak community engagement and minimal use of digital tools hinder system responsiveness and innovation capacity.

### Hospitals

Hospitals operate in isolation from the broader health system, with roles defined more by administrative levels than functional integration. Hospitals focus on curative inpatient care, neglecting essential functions such as public health, supervision, and quality improvement. Weak or non-existent referral systems contribute to overcrowding and inefficiencies, and hospitals often have insufficient mechanisms for quality assurance, mentoring, and emergency preparedness. The potential of hospitals to contribute to system-wide strengthening and integration with PHC remains untapped.

### Oversight structures

Oversight structures are constrained by centralised and fragmented governance, limiting local adaptability and accountability. Although some decentralisation exists, it is often misaligned with resources and responsibilities.^40^, ^41^, ^42^ Cross-sector coordination is poor, with minimal community involvement in governance. Health-information systems are weak, offering poor analytical capacity and feedback mechanisms. Oversight is reactive and focused on inputs rather than strategic planning or innovation, further constraining the health system’s ability to evolve and respond effectively.

## Perspective on future directions

### PCUs of the future

Primary care services are characterised by community activities, dispensaries, standalone outpatient units, health centres, clinics, and other services. PCUs offer different services, usually driven by funding or perceived disease burden. However, in the future, PCUs should provide a standardised range of first point-of-care services that meet the health and wellbeing of all individuals and families in a population. The first point-of-care interventions constitute the initial set of actions undertaken when responding to any health need. These interventions allow the beneficiary to arrest or resolve the health threat. For instance, first point-of-care interventions for malaria should include all the interventions for simple malaria, along with emergency interventions for severe malaria. To identify the first point-of-care interventions for a PCU, we first assessed the major causes of morbidity, mortality, and conditions of public health concern within the area. Based on this assessment, the required interventions to address the identified burden are determined by age cohort and across the continuum of care. Finally, the interventions that make up the first point-of-care are identified.

Instead of operating as independent service modalities, the modalities need to work together as a network providing the full range of first point-of-care services for a population. In this network model, community-based and non-facility services, including home-based care, are central and viewed as core modalities of primary care. In practice, community roles are often reduced and conflated with engagement in service delivery, rather than meaningful participation in decision making on how services are organised and delivered. Empowering communities in this way is essential for prevention, continuity of care, and person-centred PHC beyond facility-based models.

**Figure 3:**
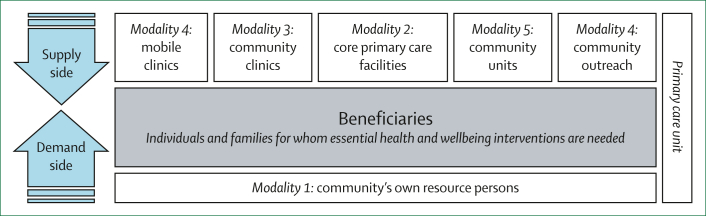
Options for service-provision modalities that constitute a primary care unit Resource persons are community members with specific skills that could be useful in supporting health-care activities. These persons could be formally trained (eg, community health workers) or untrained (eg, grandmothers or caregivers).

Individuals and families have multiple health needs, which might require interventions provided through different service-provision modalities or that extend beyond first point-of-care services. Interlinkages among service-provision modalities, along with referral capacity to link to services beyond the first point-of-care, are important for person-centred care. Each modality is best suited for specific interventions and, by working together, these modalities can expand the range of interventions provided. A PCU would determine the most appropriate modality for a situation. First point-of-care interventions for individuals and families can be provided through any of these modalities, depending on which is most efficient and effective in a specific context. Therefore, the modalities work in a complementary way to ensure the provision of all first point-of-care interventions (figure 3).

### Hospitals for the future

Currently, hospitals are viewed as providers of a broad range of clinical services, depending on their level and resource availability. We propose a more comprehensive view of hospitals, positioning them as providers of five essential services: (1) complementary services to PCUs, (2) internship services, (3) training services, (4) biomedical and public health research, and (5) clinical governance services. Although these essential services are currently provided at varying standards, the proposed framework positions these five services as the defining features of a hospital.

It is crucial to move beyond the perception that hospitals and PCUs are in competition, and instead place hospitals as a central component of future health-service delivery. Hospitals complete the care pathway and ensure the quality of primary care services through their training, internship, and clinical governance functions. Additionally, hospitals provide advanced clinical and analytical capacity to generate and support health research and analyse routine data generated by standardised health-information systems at the primary care and community levels.

The hospital sector is broad, and clear delineation of its functions is essential to ensure complementarity and prevent overlap of services. Primary hospitals, for instance, complement primary care services and complete the subnational health system, whereas higher-level hospitals focus on different specialisations. Hospitals and PCUs are complementary in their priorities (table).

**Table:** Health-service provision institutions in the WHO African region

	Primary care institution	Primary hospital institution	Secondary hospital institution	Tertiary hospital institution
Focus	First point-of-care interventions for all health needs	Interventions complementary to first point-of-care across hospital functions	Specialist interventions for specific hospital functions	Specialist interventions for all hospital functions
Outpatient unit	Outpatient routine and emergency first point-of-care interventions	Outpatient interventions complementary to first point-of-care interventions	Outpatient clinics for medicine, gynaecology, paediatrics, and surgery	Super specialist outpatient clinics
Operative unit	Outpatient procedures	Maternity theatre; emergency theatre	General theatre	Specialised theatres
Inpatient unit	Observation and stabilisation	General wards for male and female patients	Specialist wards, such as medicine, gynaecology, paediatrics and surgery, and high-dependency units	Super specialist wards and intensive care units
Investigative unit	Basic laboratory services, such as clinical chemistry and haematology	Advanced laboratory services, such as microbiology, and basic diagnostic imaging, such as ultrasound and x-ray	Specialised laboratory services, such as immunology and pathology, and advanced diagnostic radiology, such as fluoroscopy, CT, and MRI	Super-specialised laboratory services, such as molecular diagnostics; interventional radiology; and specialised diagnostic radiology, such as nuclear medicine and PET
Specialised services unit	Antenatal and maternity clinics	Specialist outpatient clinics for medicine, gynaecology, paediatrics, and surgery	Selected super specialist clinics	Autonomous super specialist clinics
Oversight unit	Facility management team and community governance team	Hospital management team, hospital board, and hospital administration		

### Health oversight for the future

By definition, health oversight involves ensuring capacity for evidence-informed, participatory decision making in the provision of essential services demanded by individuals and families.

Currently, political stakeholders and financing sources focus oversight responsibilities on building management teams, resulting in poor empowerment and engagement with actual beneficiaries beyond token engagement. Evidence should focus on minimising wastage, ensuring it is participatory, and reducing undue influence by specific stakeholders. By focusing on the legitimate needs of individuals and families, oversight functions align with a person-centred approach.

The oversight role needs to cultivate three capacities: (1) stewardship, responsible for decision making and led by the head of the oversight unit working with political and legislative health authorities; (2) management, responsible for the technical translation of decisions into actual service provision and encompassing health management teams at all levels; and (3) partnership, responsible for bringing the views and inputs of stakeholders into decision-making, planning, and monitoring processes, including coordinating committees and other partnership structures.

These capacities are necessary to guide all service-provision institutions. Oversight capacity should be strengthened in areas with deficiencies, such as primary care institutions while upholding the principle of separation of functions. Evidence from countries with substantial devolution indicates that better alignment of services with beneficiary needs correlates with more functional oversight at decentralised levels.^3^^,^^11^, ^12^, ^13^

## How to redesign primary care

The perspectives presented in this Viewpoint focus on incremental improvements in the way services should be organised, building on existing structures in the WHO African region to gradually move towards a common service-delivery architecture. This approach is practical, with adequate evidence available for implementing different factions within the region. The proposals are descriptive rather than prescriptive, outlining evolving shifts that countries can apply or adapt in diverse ways depending on the context. The vision is not to replace existing structures, but to realign functions, expectations, and roles within the system so that it becomes more responsive to population needs.

**Figure 4:**
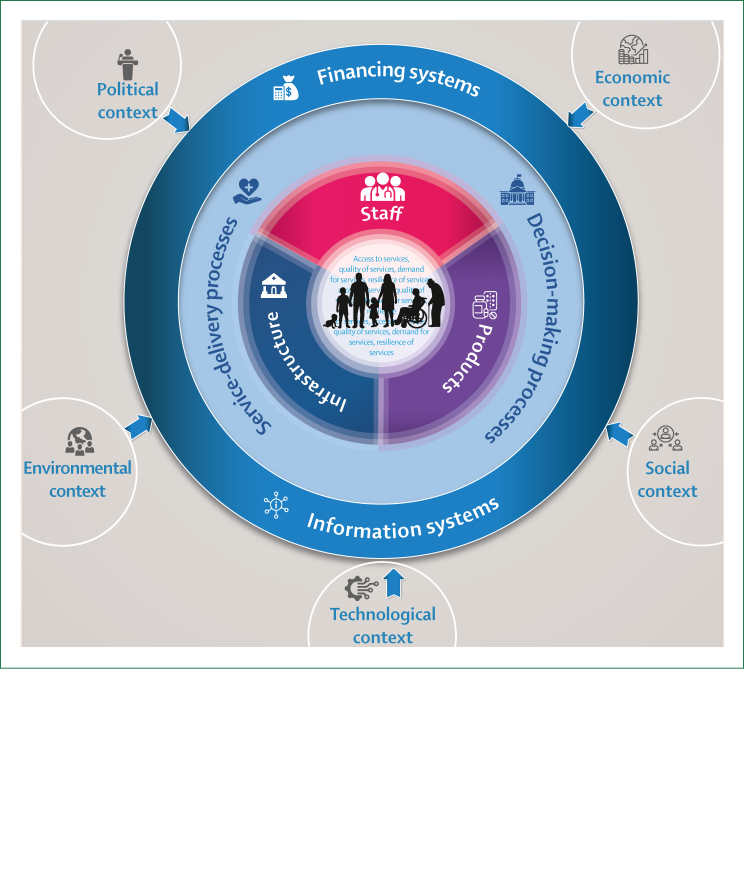
Adaptation of health-system and service integration, highlighting interlinkages and relationships from individuals and families to contextual influences

Integration in this context means aligning health-system functions of planning, financing, service provision, and monitoring people’s needs rather than focusing on disease-specific programmes. Integration requires coordination across macro (national policy and resource allocation), meso (district and subnational management), and micro (facility and community service delivery) levels. Person-centred institutions organise services around age cohorts and life-course needs, ensuring continuity of care and responsiveness according to the expectations of individuals and families (figure 4). Current institutions remain largely programme driven, resulting in fragmented service delivery and weak linkages across levels. Moving towards integration involves progressively consolidating programmes into unified service packages, supported by interoperable information systems and joint accountability mechanisms. National governments lead policy and financing reforms, subnational authorities coordinate implementation and oversight, and facilities operationalise integrated service-delivery models.

The concept of PCUs offers a framework for countries to structure first point-of-care service delivery. A well-functioning PCU ensures coordinated management across multiple service-provision modalities around common objectives to ensure the first point-of-care for everyone. Although digital innovation appears complex, it can facilitate redesign of primary care by enabling telemedicine, real-time data capture, mobile health platforms, and digital appointment systems.^43^ Primary care redesign not only expands access but also enhances the responsiveness of primary care systems to chronic conditions, demographic shifts, and sudden shock events. National governments should lead in defining and standardising PCU structures and integrating them into essential health packages, and subnational authorities should implement these models and coordinate service-provision modalities. The WHO African region should provide technical guidance and normative tools for PCU design and digital integration, and partners should support financing and innovation for telemedicine and mobile health platforms.

The proposed hospital shifts would allow better use of resources, staff, and infrastructure, ensuring that hospitals function as more than outpatient units, referral endpoints, or inpatient centres. Hospitals will help to anchor clinical quality improvement, mentor PHC providers, and support emergency preparedness. The reorientation is achievable in the short to medium term, especially in settings with ongoing decentralisation, and does not typically require costly new infrastructure. National governments should set policy directions for hospital roles and guide resource allocation, and subnational authorities should operationalise referral networks and quality improvement systems. The WHO African region should support capacity building and provide guidance on hospital governance and integration with primary care, and partners should contribute technical assistance and investments for training and emergency preparedness.

Finally, the oversight capacities provide greater direction, coordination, and accountability within the sector, with an emphasis on real-time, evidence-based decision making.^44^ Such capacities enable real participatory governance through practical channels for citizen input, local priority setting, and social accountability. Oversight institutions such as health-facility committees, district health assemblies, and regular public performance reviews can help to build legitimacy and trust. Digital platforms that focus on supporting decision making, and not compliance reporting alone, will facilitate this.^32^ Managers require control over key inputs but should remain accountable for results. Such reconfiguration is possible through policy reforms and capacity-building efforts, building on existing decentralisation and service-integration processes. Ultimately, these changes enable real-time, operational-level decision making aligned with population needs. National governments should strengthen policy frameworks for participatory governance, and subnational authorities should implement oversight mechanisms and ensure accountability. Technical support is needed for governance reforms and digital platforms, and partners should assist with resources and innovation for social accountability tools.

Several initiatives can support countries in the re-alignment of health-service provision. National governments should lead efforts to redefine a fit-for-purpose district health system, providing a menu of options for planning, organisation, and management of health services at the primary care, hospital, and oversight levels. Additionally, national governments should develop a health managers’ guide of processes to operationalise the proposed recommendations, ensuring that subnational authorities have the tools for implementation. Subnational authorities should focus on applying these tools and scaling up the use of updated essential health packages to clarify interventions by age cohort and across the continuum of care. Technical guidance and templates are needed to rationalise the use of different health-service provision modalities and support countries in identifying the most appropriate modalities for specific contexts. In addition, policy laboratories should be used to coordinate periodic reviews, share lessons learnt, and navigate implementation challenges while ensuring that these platforms remain representative and technically balanced. Partners should assist with financing and innovation to maximise the potential role of digital solutions, including artificial intelligence, as key enablers of cost-efficient expansion of access to, and quality of, care for individuals and families.

## Strengths and limitations

A key strength of this Viewpoint is its region-wide scope, grounded in multicountry evidence and expert consensus on implementing the revitalised PHC approach. The process drew on structured methodologies (ie, nominal group technique and Delphi) to ensure inclusivity and reduce the dominance of individual views.

Limitations of this Viewpoint include the absence of empirical validation of the proposed pathways and reliance on system-level perspectives, which might not fully capture operational dynamics at facility or community levels. In addition, although efforts were made to ensure diverse expert representation across the three policy laboratories, we recognise that some areas of expertise might have been under-represented. Future research should focus on country case studies and empirical analyses to refine and test the proposed pathways in diverse contexts.

## Conclusion

**Figure 5:**
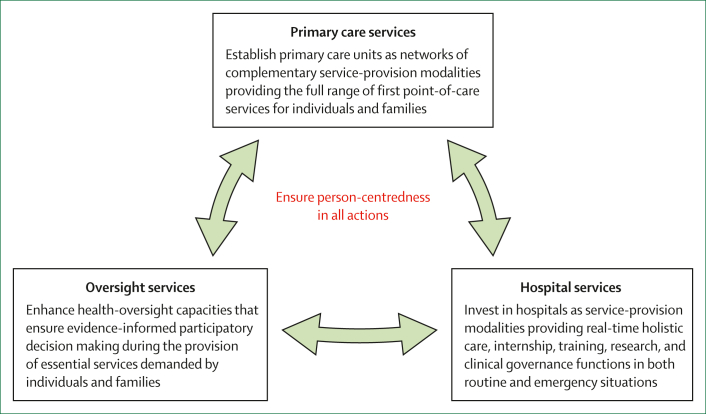
Overall shifts for the future of health-service delivery in the WHO African region

The future calls for reframing the provision of essential services as integrated, person-centred, and interconnected institutions working together to maximise health and wellbeing. Most countries will need phased investments and adaptations shaped by political and financial realities. However, we argue that considerable gains can be achieved by reorganising existing resources into functional units. We summarise the recommendations under three overarching and interrelated themes, all focused on the need for person-centred health services (figure 5).

We need to redesign Africa’s future health-service delivery to meet the population needs, withstand shocks, and leverage technological opportunities. In this Viewpoint, we do not focus on replacing existing structures and investments, but on repurposing and realigning them.

National and subnational governments should lead these changes, with support from regional and global partners. Investments, policies, and innovations should align with the goal of resilient, people-centred health systems. The path forward is collective, and a shared commitment will shape what health systems in the WHO African region can deliver in the decades ahead.

## Declaration of interests

We declare no competing interests.

## References

[1] Karamagi H, Iwu-Jaja C, Mazingisa AV (2026). A scoping review of African health histories from the pre-colonial to SDG eras: insights for future health systems. Healthcare.

[2] Rifkin SB (2018). Alma Ata after 40 years: primary health care and health for all–from consensus to complexity. BMJ Glob Health.

[3] Walsh JA, Warren KS (1979). Selective primary health care: an interim strategy for disease control in developing countries. N Engl J Med.

[4] Ebrahim GJ (1993). The Bamako initiative. J Trop Pediatr.

[5] Knippenberg R, Alihonou E, Soucat A (1997). Implementation of the Bamako Initiative: strategies in Benin and Guinea. Int J Health Plann Manage.

[6] Lea RA (1993). World development report 1993: ‘investing in health’. Forum Dev Stud.

[7] WHO African Region (2022). Atlas of African health statistics 2022: health situation analysis of the WHO African region. https://iris.who.int/handle/10665/364851.

[8] WHO Evaluation Office (December 2019). Review of 40 years of primary health care implementation at country level. https://www.who.int/docs/default-source/documents/about-us/evaluation/phc-final-report.pdf.

[9] Moyo E, Mhango M, Moyo P, Dzinamarira T, Chitungo I, Murewanhema G (2023). Emerging infectious disease outbreaks in sub-Saharan Africa: learning from the past and present to be better prepared for future outbreaks. Front Public Health.

[10] UN (2024). World population prospects 2024. Summary of results. https://population.un.org/wpp/assets/Files/WPP2024_Summary-of-Results.pdf.

[11] WHO Regional Office for Africa (2018). The state of health in the WHO African Region: an analysis of the status of health, health services and health systems in the context of the sustainable development goals. https://www.afro.who.int/sites/default/files/sessions/documents/State%20of%20health%20in%20the%20African%20Region.pdf.

[12] Bitton A, Ratcliffe HL, Veillard JH (2017). Primary health care as a foundation for strengthening health systems in low- and middle-income countries. J Gen Intern Med.

[13] Gilson L, Barasa E, Nxumalo N (2017). Everyday resilience in district health systems: emerging insights from the front lines in Kenya and South Africa. BMJ Glob Health.

[14] WHO & UNICEF (Feb 28, 2022). Primary health care measurement framework and indicators: monitoring health systems through a primary health care lens. https://www.who.int/publications/i/item/9789240044210.

[15] Karamagi HC, Tumusiime P, Titi-Ofei R (2021). Towards universal health coverage in the WHO African region: assessing health system functionality, incorporating lessons from COVID-19. BMJ Glob Health.

[16] Mash R (2018). The Astana Declaration and future African primary health care. Afr J Prim Health Care Fam Med.

[17] Rivas-Morello B, Horemans D, Viswanathan K (2023). Assessing capacities and resilience of health services during the COVID-19 pandemic: lessons learned from use of rapid key informant surveys. Front Public Health.

[18] Reeve C, Humphreys J, Wakerman J (2015). Community participation in health service reform: the development of an innovative remote Aboriginal primary health-care service. Aust J Prim Health.

[19] McMillan SS, Kelly F, Sav A (2014). Using the nominal group technique: how to analyse across multiple groups. Health Serv Outcomes Res Method.

[20] Nasa P, Jain R, Juneja D (2021). Delphi methodology in healthcare research: how to decide its appropriateness. World J Methodol.

[21] Ahmad M, Wilkins S (2025). Purposive sampling in qualitative research: a framework for the entire journey. Qual Quant.

[22] WHO Regional Office for Africa (2024). The future of health service delivery in the context of attaining universal health coverage, health security, and healthier populations in the African region. https://www.afro.who.int/sites/default/files/2026-01/The%20Future%20of%20Health%20Service%20Delivery%20in%20the%20African%20Region%20Health%20Policy%20Laboratories%20Report..%20.pdf.

[23] WHO (May 15, 2025). World health statistics 2025: monitoring health for the SDGs, sustainable development goals. https://iris.who.int/bitstream/handle/10665/381418/9789240110496-eng.pdf?sequence=1.

[24] Iwu-Jaja C, Iwu CD, Jaca A, Wiysonge CS (2023). New vaccine introductions in WHO African region between 2000 and 2022. Vaccines (Basel).

[25] Karamagi HC, Berhane A, Ngusbrhan Kidane S (2022). High impact health service interventions for attainment of UHC in Africa: a systematic review. PLoS Glob Public Health.

[26] Karamagi HC, Kidane SN, Kariyo PC (2025). Proceedings of essential health care package development, in Botswana and Sierra Leone, November 2022. BMC Proc.

[27] Ahmat A, Asamani JA, Abdou Illou MM (2022). Estimating the threshold of health workforce densities towards universal health coverage in Africa. BMJ Glob Health.

[28] Asamani JA, Bediakon KSB, Boniol M (2024). State of the health workforce in the WHO African region: decade review of progress and opportunities for policy reforms and investments. BMJ Glob Health.

[29] Karamagi HC, Afriyie DO, Ben Charif A (2024). Mapping inequalities in health service coverage in Africa: a scoping review. BMJ Open.

[30] Oke GI, Sibomana O (2025). Understanding health inequality, disparity and inequity in Africa: a rapid review of concepts, root causes, and strategic solutions. Public Health Chall.

[31] Alaran MA, Lawal SK, Jiya MH (2025). Challenges and opportunities of artificial intelligence in African health space. Digit Health.

[32] Ibeneme S, Karamagi H, Muneene D, Goswami K, Chisaka N, Okeibunor J (2022). Strengthening health systems using innovative digital health technologies in Africa. Front Digit Health.

[33] Karamagi HC, Muneene D, Droti B (2022). EHealth or e-Chaos: the use of digital health interventions for health systems strengthening in sub-Saharan Africa over the last 10 years: a scoping review. J Glob Health.

[34] Olu OO, Petu A, Usman A (2024). Leaving no one behind in armed conflict-affected settings of Africa: is universal health coverage a possibility or mirage?. Glob Health Res Policy.

[35] Nweke OC, Sanders WH (2009). Modern environmental health hazards: a public health issue of increasing significance in Africa. Environ Health Perspect.

[36] Tumusiime P, Karamagi H, Titi-Ofei R (2020). Building health system resilience in the context of primary health care revitalization for attainment of UHC: proceedings from the Fifth Health Sector Directors’ policy and planning meeting for the WHO African region. BMC Proc.

[37] Karamagi HC, Titi-Ofei R, Kipruto HK (2022). On the resilience of health systems: a methodological exploration across countries in the WHO African region. PLoS One.

[38] Tumusiime P, Nabyonga-Orem J, Karamagi H (2019). Resilient health systems for attaining universal health coverage. BMJ Glob Health.

[39] Karamagi H, Titi-Ofei R, Amri M (2022). Cross country lessons sharing on practices, challenges and innovation in primary health care revitalization and universal health coverage implementation among 18 countries in the WHO African region. Pan Afr Med J.

[40] Masaba BB, Moturi JK, Taiswa J, Mmusi-Phetoe RM (2020). Devolution of healthcare system in Kenya: progress and challenges. Public Health.

[41] McCollum R, Theobald S, Otiso L (2018). Priority setting for health in the context of devolution in Kenya: implications for health equity and community-based primary care. Health Policy Plan.

[42] Kesale AM, Mahonge C, Muhanga M (2022). Effects of decentralization on the functionality of health facility governing committees in lower and middle-income countries: a systematic literature review. Glob Health Action.

[43] Wootton R (1998). Telemedicine in the national health service. J R Soc Med.

[44] Bigdeli M, Rouffy B, Lane BD, Schmets G, Soucat A (2020). Health systems governance: the missing links. BMJ Glob Health.

