# Supplementary material for: Organisation of health services for the delivery of primary health care in the WHO African region: a future perspective
Source: Lancet Prim Care. 2026 Apr;2(4):None. doi: 10.1016/j.lanprc.2026.100122 (PMC13153028; doi:10.1016/j.lanprc.2026.100122)
Supplement: Supplementary appendix [file mmc1.pdf]

THE LANCET
Primary Care

Supplementary appendix

This appendix formed part of the original submission and has been peer reviewed. We post it as supplied by the authors.

Supplement to: Karamagi H, Mamo A, Droti B, et al. Organisation of health services for the delivery of primary health care in the WHO African region: a future perspective. *Lancet Prim Care* 2026. https://doi.org/10.1016/j.lanprc.2026.100122

Table of content

Appendix 1: Experts that participated in the study 2
Appendix 2: Semi structured Questionnaire 3
POLICY LAB 1: PRIMARY CARE UNITS __________ 4
POLICY LAB 2: HOSPITALS (3 tools: primary, secondary, tertiary) 6
POLICY LAB 3: OVERSIGHT AND MANAGEMENT TEAMS (3 tools- sub national, regional, national) 8
Appendix 3: Health System Performance Index (2021) 10
References 11

Appendix 1: Experts that participated in the study

Policy Lab 1		Category	Policy Lab 2		Category	Policy Lab 3		Category
Adionilde Aguilar	STP	Expert, practitioner	Alberta Biritwum Nyarko	Ghana	Expert, Practitioner	Kedieinde Akec Chong Mapour	South Sudan	Expert
Caroline Omar	Mozambique		Aruasa Wilson	Kenya		Andrew Mulwa	Kenya	Academia
Christine Mataza	Kenya	Practitioner	Dr Bonevanture Nzeyimana	Rwanda	Expert	Anthony Ofosu	Ghana	Practitioner, Expert and Advocate
Edward Mumbo	Kenya	Expert	Dr Isaulina Barreto	STP	Practitioner	Araia Berhane	Eritrea	Expert
Eusebio Chaquisse	Mozambique	Expert	Dr Terence Carter	South Africa	Expert	Benjamin Wonder Kwesi Nyakutsey	Ghana Advocate	
Evgeny Zheleznyakov	STP	Advocate	Dr.Tadjidine Youssouf	Comoros	Practitioner	Darius Uzabakiriho	Rwanda	Expert
Gilbert Abotisem Abiiro	Ghana	Expert	Evelyne Ryumeko	Burundi	Advocate	Delanyo Dovlo	Ghana	Expert
John Rumunu	South Sudan	Expert	Gaci Boualem	Algeria	Expert	Eduardo Samo Gudo	Mozambique	Practitioner
Kahsu Bekuretsion	Ethiopia	Expert	Gilbert Buckle,	Ghana	Expert	Elizabeth Wangia	Kenya	Practitioner, Expert
Kouame Kobenan Kouman Arnold	Côte D'ivoire	Expert, Practitioner, Academic or Advocate	Grim Nasser	Algeria	Practitioner	Freddie Sengooba	Uganda Expert	
Nicimpaye Anglebert	Burundi	Practitioner, Expert	Iqbal Khandwalla	Kenya	Advocate	Gashubije Longin	Burundi	Expert
Paul Kagwa	Uganda	Advocate	Isabella Maina	Kenya	Practitioner	Gordon Abekah-Nkrumah	Ghana	Academia
Peter Agyei-Baffour	Ghana	Advocate	José Cunha	Angola	Practitioner	Henry Kansembe	Zambia	Expert
Poda Gnimbar Ghislain	Burkina Faso	Expert, Practitioner, Academic	Lee Wallis	South Africa	Academia	Humberto Muquingue	Mozambique	- Advocate
Ramos Bartolomeu Mboane	Mozambique	Practitioner	Maria Dos Prazeres	Mozambique	Academia	Julie Jemutai	Kenya	Academic and expert
Teresa Kinyari	Kenya	Academic	Nicayenzi Dieudonné	Burundi	Practitioner, Expert	Marin Kokou Wotobe	Togo	Advocate
			Patience Aseweh Abor	Ghana	Acadamia	Marion Okoh-Owusu	Ghana	Practitioner
			Peter Agyei-Baffour	Ghana	Academia	Roger A. Atinga	Ghana	Academia/Researcher
			Takbou Idir	Algeria	Advocate	Saidou Pathe Barry	Guinea	Expert
						Salmi Madjid	Algeria	Academic
						Sara Cordeiro Pereira	STP	Practitioner, Academic
						Soraya Elloker	South Africa	Academic
						Yogan Pillay	South Africa	Expert

Submission	16			Submission	19			Submission	23	

Legend	
Green and White	Experts that participated in step 2 and 4
Green	Experts that participated in step 2, 4 and 5

Appendix 2: Semi structured Questionnaire

Development and piloting of the semi structured questionnaire for Step 2

The semi structured questionnaire used in Step 2 was drawn from a tool previously applied in the State of Health in Africa assessment (1), where it had been piloted and validated through its prior use for collecting qualitative information on health service organization across age cohorts and system domains. This provided the areas of assessment in the tool.

The four areas for which information was derived were aligned with strategic assessment areas used in organizational review processes: practices, bottlenecks, future options and aspirations (2 – 4).

Prior to application, the questionnaire was presented and discussed with the key informants during a virtual briefing session to ensure consistent understanding of its content and purpose.

Approach to qualitative data consolidation for Step 3

The qualitative data analysis followed a structured thematic process. Inputs from experts were all collated based on the four thematic areas: practices, bottlenecks, future options and aspirations. All information from the key informant experts was captured based on the areas. From the data, the emerging themes for which there was consistent data were around: (1) a definitional summary of the policy laboratory; (2) what services it should provide; (3) core inputs needed; and (4) what is needed to make the laboratory area ready for transformation. For each of these, there were options that arose from the different experts. These formed the thematic summaries for each policy laboratory that were used in subsequent steps.

Consensus building methodology for Steps 4 and 5

In Step 4, the thematic summary for each policy laboratory was shared with all experts for review and validation. They reviewed to confirm whether the summaries represented their views, and if there were some additional aspects they had omitted. An updated set of options for each policy laboratory was therefore arrived at, incorporating the views of all the experts.

In Step 5, the experts met physically for five days to determine consensus around the consolidated options. All the experts were invited, with 31 out of 58 eventually attending. At the meeting, the experts for each laboratory reviewed the options in each of the emerging themes. The Nominal Group Technique was used to identify areas where views diverged by each policy laboratory. The three policy laboratories convened for peer review of each others outputs to identify any potential missed areas, and inter-connections across the policy labs. Following this, each policy laboratory re-convened to build consensus around single set of descriptions in each of their thematic areas, using the Delphi technique. Three rounds were targeted – each round constituting a discussion within the policy laboratory, followed by a consensus discussion involving all the policy laboratories. Consensus results were achieved for thematic areas 2 and 3 (areas of focus and inputs) after the 1st Delphi round, for area 4 (aspirations) after the 2nd round, with area 1 (definitions) eventually achieving consensus at discussions following the 3rd Deplhi round.

POLICY LAB 1: PRIMARY CARE UNITS

Role	Area of assessment		PRACTICE What is the current practice?	BOTTLENECKS What are the key challenges experienced	ASPIRATION What needs to exist, for this to contribute to UHC, HSE and DOH outcomes	OPTIONS What needs to be done, to move towards the needed capacity	Additional comments
**Service provision**	First point of care for services of promotion, prevention, diagnostic, curative, rehabilitative, & palliative services	Pregnancy and newborn age cohort					
		Childhood age cohort					
		Adolescent age cohort					
		Adult age cohort					
		Elderly age cohort					
	Provision of services through different modalities	Fixed health facilities					
		Outreach units					
		Mobile community-based clinics					
		Community units taking services into the community					
		Digital services					
	Specialized services	Emergency care services including outpatient operative care and first aid					
**Management (sub district team)**	Structure	How resources (HRH, infrastructure, products) are organized to deliver expected results					
	Strategy	The approach to achieving expected results					
	System	Processes and workflows to accomplish planned activities					
	Style	Informal rules and cultures that define how it works					
	Staff	How each employee develops and grows in their roles					
	Skills	Expertise and competencies expected to deliver on results					
	Shared values	Principles and standards of behavior everyone is expected to adhere to					

	Resource availability & adequacy	Public funding					
		External funding					
		Private sector funding					
		Voluntary contributions					
		Household / out of pocket funding					
	Resource management	Budget priority setting process					
		Efficiency in resource utilization					
		Transparency and participation of beneficiaries / users					
		Budget control and audit					
**Oversight (respective authority)**	Authority & mandate	Where decision making power is drawn from					
	Organizational structure	Formal and information organograms					
	Policy / strategic guidance	The planning process, and documents available					
	Laws and regulations	The rules of the game					
	Social accountability	How the system reports to beneficiaries					
	Technical accountability	How to system reports to partners and funding sources					
	Beneficiary involvement	How beneficiaries and other local actors are involved in implementation oversight					
**Coordination**	With other public institutions	Engagement with other social sectors (Education, Water, etc)					
		Engagement with Finance and Planning Ministries					
		Engagement with disaster coordination actors					
	With non-public institutions	Engagement with civil society					
		Engagement with private for-profit actors					
		Engagement with not-for-profit actors					
	With external partners	Engagement with donor agencies					
		Engagement with technical partners					

		Engagement with NGOs / implementing partners					

POLICY LAB 2: HOSPITALS (3 tools: primary, secondary, tertiary)

Role	*Area of assessment*		*PRACTICE What is the current practice?*	*BOTTLENECKS What are the key challenges experienced*	*ASPIRATION What needs to exist, for this to contribute to UHC, HSE and DOH outcomes*	*OPTIONS What needs to be done, to move towards the needed capacity*	*Additional comments*
**Service provision**	Specialist services of promotion, prevention, diagnostic, curative, rehabilitative, & palliative services	Pregnancy and newborn age cohort					
		Childhood age cohort					
		Adolescent age cohort					
		Adult age cohort					
		Elderly age cohort					
	Provision of services through different modalities	24-hour outpatient services					
		24-hour inpatient services					
		24-hour routine operative services					
		Specialist in facility clinics					
		Specialist camps in lower facilities / communities					
		Digital / telemedicine services					
		Critical care services:<br>▪ 1st level hospitals – HDUs<br>▪ 2nd level hospitals - general ICUs<br>▪ 3rd level hospitals - Specialist ICUs					
	Specialized services	Pre-service training					
		Internship services					
		Research services					
		Oversight and support to clinical services in lower-level facilities (e.g. technical supervision, specialist clinics, specialist camps, etc)					
	Structure	How resources (HRH, infrastructure, products) are					

**Management (Hospital Mgt Team)**		organized to deliver expected results					
	Strategy	The approach to achieving expected results					
	System	Processes and workflows to accomplish planned activities					
	Style	Informal rules and cultures that define how it works					
	Staff	How each employee develops and grows in their roles					
	Skills	Expertise and competencies expected to deliver on results					
	Shared values	Principles and standards of behavior everyone is expected to adhere to					
	Resource availability & adequacy	Public funding					
		External funding					
		Private sector funding					
		Voluntary contributions					
		Household / out of pocket funding					
	Resource management	Budget priority setting process					
		Efficiency in resource utilization					
		Transparency and participation of beneficiaries / users					
		Budget control and audit					
**Oversight (respective authority)**	Authority & mandate	Where decision making power is drawn from					
	Organizational structure	Formal and information organograms					
	Policy / strategic guidance	The planning process, and documents available					
	Laws and regulations	The rules of the game					
	Social accountability	How the system reports to beneficiaries					
	Technical accountability	How to system reports to partners and funding sources					
	Beneficiary involvement	How beneficiaries and other local actors are involved in implementation oversight					
**Coordination**	With other public institutions	Engagement with other social sectors (Education, Water, etc)					
		Engagement with Finance and Planning Ministries					
		Engagement with disaster coordination actors					
		Engagement with civil society					

	With non-public institutions	Engagement with private for-profit actors					
		Engagement with not-for-profit actors					
	With external partners	Engagement with donor agencies					
		Engagement with technical partners					
		Engagement with NGOs / implementing partners					

POLICY LAB 3: OVERSIGHT AND MANAGEMENT TEAMS (3 tools - sub national, regional, national)

Role	Area of assessment		PRACTICE *What is the current practice?*	BOTTLENECKS *What are the key challenges experienced*	ASPIRATION *What needs to exist, for this to contribute to UHC, HSE and DOH outcomes*	OPTIONS *What needs to be done, to move towards the needed capacity*	Additional comments
**Management**	Structure	How resources (HRH, infrastructure, products) are organized to deliver expected results					
	Strategy	The approach to achieving expected results					
	System	Processes and workflows to accomplish planned activities					
	Style	Informal rules and cultures that define how it works					
	Staff	How each employee develops and grows in their roles					
	Skills	Expertise and competencies expected to deliver on results					
	Shared values	Principles and standards of behavior everyone is expected to adhere to					
	Resource availability & adequacy	Public funding					
		External funding					
		Private sector funding					
		Voluntary contributions					
		Household / out of pocket funding					
	Resource management	Budget priority setting process					
		Efficiency in resource utilization					
		Transparency and participation of beneficiaries / users					
		Budget control and audit					
	Authority & mandate	Where decision making power is drawn from					

**Oversight**	Organizational structure	Formal and information organograms					
	Policy / strategic guidance	The planning process, and documents available					
	Laws and regulations	The rules of the game					
	Social accountability	How the system reports to beneficiaries					
	Technical accountability	How to system reports to partners and funding sources					
	Beneficiary involvement	How beneficiaries and other local actors are involved in implementation oversight					
**Coordination**	With other public institutions	Engagement with other social sectors (Education, Water, etc)					
		Engagement with Finance and Planning Ministries					
		Engagement with disaster coordination actors					
	With non-public institutions	Engagement with civil society					
		Engagement with private for-profit actors					
		Engagement with not-for-profit actors					
	With external partners	Engagement with donor agencies					
		Engagement with technical partners					
		Engagement with NGOs / implementing partners					

Appendix 3: Health System Performance Index (2021)

Country	Health System Performance Index (2021)
Algeria	67.7
Angola	48.8
Burkina Faso	57.2
Burundi	49.8
Comoros	52.4
Côte d'Ivoire	48.6
Eritrea	50.2
Ethiopa	44.8
Ghana	58.2
Guinea	46.9
Kenya	62.5
Mozambique	58.2
Rwanda	52
Sao Tome and Principe	56.6
South Africa	71.5
South Sudan	42.4
Togo	44.8
Uganda	58.3
Zambia	59

Source: Towards universal health coverage in the WHO African Region (5)

References

1. World Health Organization Regional Office for Africa. *State of health in the WHO African Region.* [Internet]. Brazzaville; 2018 [cited 2026 Jan 16]. Available from: https://www.afro.who.int/publications/state-health-who-african-region

2. Lusthaus C, Adrien M-H. *Organizational Assessment: A Review of Experience.* [Internet]. Montreal: Universalia; 1998 [cited 2026 Jan 16]. Available from: https://www.universalia.com/sites/default/files/articles/fichiers/no31_oaexperiencereview.pdf

3. Shinkle GA. Organizational aspirations, reference points, and goals: Building on the past and aiming for the future. *Journal of Management.* [Internet]. 2012;38(1):415-455 [cited 2026 Jan 16]. Available from: https://journals.sagepub.com/doi/abs/10.1177/0149206311419856

4. Korman AK. Hypothesis of work behavior revisited and an extension. *Academy of Management Review.* [Internet]. 1976;1(1):50-64 [cited 2026 Jan 16]. Available from: https://journals.aom.org/doi/10.5465/amr.1976.4408670

5. Karamagi HC, Tumusiime P, Titi-Ofei R, Droti B, Kipruto H, Nabyonga-Orem J, et al. Towards universal health coverage in the WHO African Region: assessing health system functionality, incorporating lessons from COVID-19. BMJ Glob Health [Internet]. 2021 Mar 31 [cited 2025 Sep 9];6(3):4618. Available from: https://gh.bmj.com/content/6/3/e004618
